# COVID-19 Vaccination Coverage Among Adults — United States, December 14, 2020–May 22, 2021

**DOI:** 10.15585/mmwr.mm7025e1

**Published:** 2021-06-25

**Authors:** Jill Diesel, Natalie Sterrett, Sharoda Dasgupta, Jennifer L. Kriss, Vaughn Barry, Kayla Vanden Esschert, Ari Whiteman, Betsy L. Cadwell, Daniel Weller, Judith R. Qualters, LaTreace Harris, Achal Bhatt, Charnetta Williams, LeAnne M. Fox, Dana Meaney Delman, Carla L. Black, Kamil E. Barbour

**Affiliations:** ^1^CDC COVID-19 Response Team; ^2^Michigan Department of Health and Human Services; ^3^Epidemic Intelligence Service, CDC; ^4^Geospatial Research, Analysis, and Services Program, Agency for Toxic Substances and Disease Registry, Atlanta, Georgia.

The U.S. COVID-19 vaccination program launched on December 14, 2020. The Advisory Committee on Immunization Practices recommended prioritizing COVID-19 vaccination for specific groups of the U.S. population who were at highest risk for COVID-19 hospitalization and death, including adults aged ≥75 years*; implementation varied by state, and eligibility was gradually expanded to persons aged ≥65 years beginning in January 2021. By April 19, 2021, eligibility was expanded to all adults aged ≥18 years nationwide.^†^ To assess patterns of COVID-19 vaccination coverage among U.S. adults, CDC analyzed data submitted on vaccinations administered during December 14, 2020–May 22, 2021, by age, sex, and community-level characteristics. By May 22, 2021, 57.0% of persons aged ≥18 years had received ≥1 COVID-19 vaccine dose; coverage was highest among persons aged ≥65 years (80.0%) and lowest among persons aged 18–29 years (38.3%). During the week beginning February 7, 2021, vaccination initiation among adults aged ≥65 years peaked at 8.2%, whereas weekly initiation among other age groups peaked later and at lower levels. During April 19–May 22, 2021, the period following expanded eligibility to all adults, weekly initiation remained <4.0% and decreased for all age groups, including persons aged 18–29 years (3.6% to 1.9%) and 30–49 years (3.5% to 1.7%); based on the current rate of weekly initiation (as of May 22), younger persons will not reach the same levels of coverage as older persons by the end of August. Across all age groups, coverage (≥1 dose) was lower among men compared with women, except among adults aged ≥65 years, and lower among persons living in counties that were less urban, had higher social vulnerabilities, or had higher percentages of social determinants of poor health. Continued efforts to improve vaccination confidence and alleviate barriers to vaccination initiation, especially among adults aged 18–49 years, could improve vaccination coverage.

Vaccination data were reported to CDC via state immunization information systems,^§^ the Vaccine Administration Management System,^¶^ or direct data submission to the CDC Data Clearinghouse.** Data for vaccinations administered among adults aged ≥18 during December 14, 2020–May 22, 2021, were included in the analysis.^††^ Two measures of vaccination coverage were assessed: 1) persons who received ≥1 dose of any COVID-19 vaccine (≥1-dose coverage) authorized by the Food and Drug Administration (FDA) and 2) persons who received 2 doses of an FDA authorized 2-dose vaccine (Pfizer-BioNTech or Moderna) or 1 dose of the Janssen (Johnson & Johnson) vaccine (fully vaccinated); each measure of coverage was calculated using total population counts from the U.S. Census Bureau’s 2019 Population Estimates Program.^§§^ Weekly vaccine initiation was defined as the percentage of persons who received the first dose within the epidemiologic week^¶¶^ among those in the total population. Coverage (≥1 dose) was projected through the week of August 29, 2021, by applying the rate of weekly initiation in the most recent week (May 22) for each age group to subsequent weeks beyond the study period. Second dose completion was defined as the percentage of persons who received the second dose of a 2-dose vaccine at any point, among those who had received at least 1 dose of a 2-dose vaccine.*** Absolute differences in coverage by age were calculated during three periods selected to represent general shifts in targeted subpopulations, supply, and policy over the course of the COVID-19 vaccination program^†††^ (*1*): December 14, 2020–January 23, 2021; January 24, 2021–March 20, 2021; and March 21, 2021–May 22, 2021.

Coverage was evaluated by selected community-level characteristics matched to vaccine recipients’ county of residence.^§§§^ County-level rankings of social vulnerability from the 2018 CDC Social Vulnerability Index (SVI), which is used to identify community needs during emergencies, were categorized into quartiles based on distribution among all U.S. counties.^¶¶¶^ County-level data on Social Determinants of Health**** obtained from the American Community Survey^††††^ were dichotomized based on the median of all U.S. counties.^§§§§^ County-level urbanicity was based on the 2013 National Center for Health Statistics urban-rural classification scheme.^¶¶¶¶^ Generalized estimating equation models with binomial regression and an identity link were used to estimate absolute differences in coverage and associated 95% confidence intervals. SAS (version 9.4; SAS Institute) was used to conduct all analyses. This activity was reviewed by CDC and was conducted consistent with applicable federal law and CDC policy.*****

During December 14, 2020–May 22, 2021, 57.0% of U.S. adults had received ≥1 vaccine dose; coverage was highest among adults aged ≥65 years (80.0%) and lowest among adults aged 18–29 years (38.3%) (Figure 1). Vaccination coverage was lower among younger age groups in all states, regardless of timing of expanded vaccine eligibility to all adults (Supplementary Table, https://stacks.cdc.gov/view/cdc/107123). During January 24, 2021–March 20, 2021, coverage among persons aged ≥65 years increased from 14.3% to 67.0% (absolute difference: 52.7%). During March 21, 2021–May 22, 2021, absolute increases in coverage were largest among adults aged 50–64 years (31.5% to 63.5%; absolute difference: 32.0%).

**FIGURE 1:**
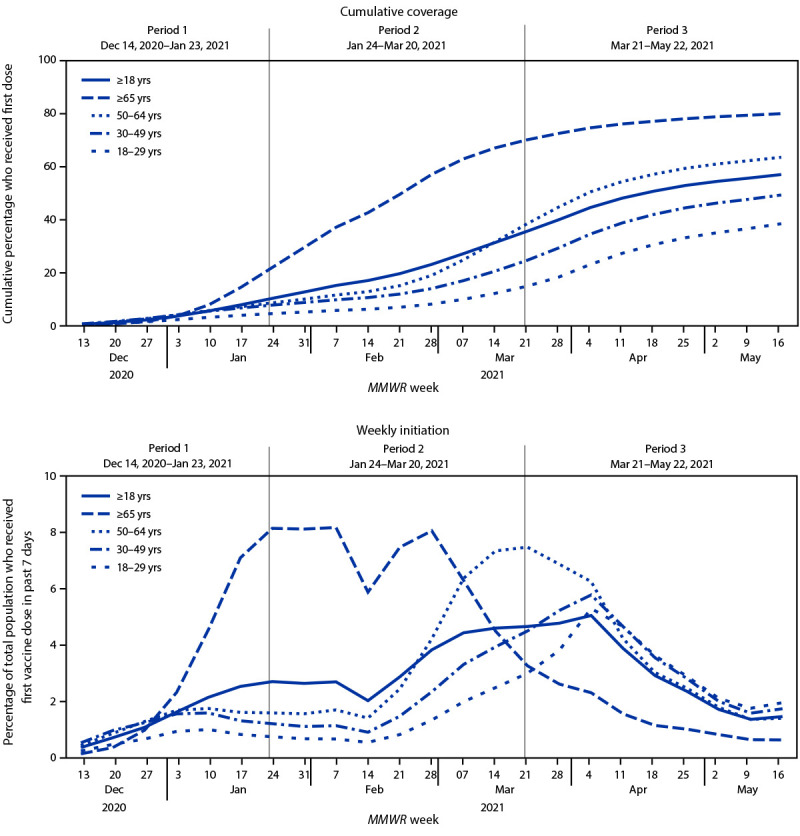
Trends in COVID-19 vaccination cumulative coverage* and weekly initiation among adults, by epidemiologic week^†^ and age group — United States, December 14, 2020–May 22, 2021 * Coverage includes persons who received at least 1 dose of any Food and Drug Administration–authorized COVID-19 vaccine (≥1 dose; Pfizer-BioNTech, Moderna, or Janssen [Johnson & Johnson]). ^†^ An epidemiologic week is based on the National Notifiable Diseases Surveillance System guidance and is assigned by the reporting local or state health department for the purposes of *MMWR* disease incidence reporting and publishing. The first day of any *MMWR* week is Sunday. https://wwwn.cdc.gov/nndss/document/MMWR_Week_overview.pdf

Over the entire period, weekly initiation was highest among adults aged ≥65 years and peaked during the week of February 7, during which 8.2% of adults aged ≥65 years initiated vaccination (Figure 1). Weekly initiation peaked at 7.5% among adults aged 50–64 years during the week of March 21, at 5.8% among adults aged 30–49 years during the week of April 4, and at 5.3% among adults aged 18–29 years during the week of April 4. Since the week of April 18, during which eligibility was expanded to all adults, weekly COVID-19 vaccine initiation was <4.0% and decreased over time for all age groups, including younger adults aged 18–29 years (3.6% to 1.9%) and 30–49 years (3.5% to 1.7%). If weekly initiation remains at the rate as of the week of May 22 for each age group, coverage by the week of August 29, 2021 is projected to reach 57.5% for adults aged 18–29 years, 71.4% for adults aged 30–49 years, 85.9% for adults aged 50–64 years, 94.9% for adults aged ≥65 years, and 78.4% for persons aged ≥18 years.

By May 22, among adults who initiated a 2-dose vaccine series (Pfizer-BioNTech or Moderna), 89.3% had received their second dose at any point. The second dose completion was similar across age groups (Figure 2) and over time.

**FIGURE 2:**
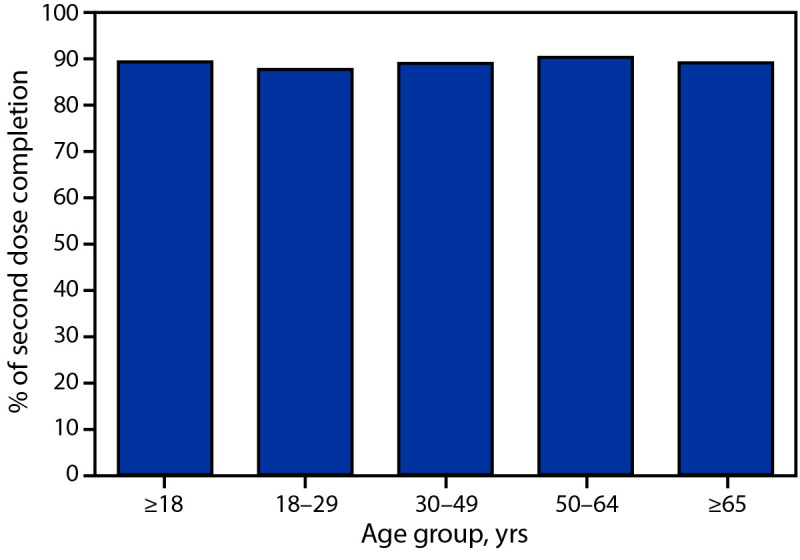
COVID-19 vaccination second dose completion among adults who received ≥1 COVID-19 dose and had sufficient time to receive the second dose,* by age group — United States,^†^ December 14, 2020–May 22, 2021 * Analysis for second dose completion was restricted to persons who had received their first dose of a 2-dose vaccine (Pfizer-BioNTech or Moderna) during December 14, 2020–March 31, 2021. All persons included in the analysis for second dose completion were ≥42 days past their first dose. ^†^ Excludes residents of Texas because Texas does not report information for age-specific dose number to CDC.

Men had lower coverage than women in all age groups, except those aged ≥65 years (Table). Persons living in counties that were less urban were less likely to be vaccinated, and differences were smaller for adults aged ≥65 years. Across all age groups, people living in counties with higher social vulnerabilities or higher percentages of the population who are uninsured, living in poverty, lacking access to a computer, and lacking access to a computer with Internet were less likely to be vaccinated.

**TABLE:** Coverage with ≥1 dose COVID-19 vaccine* among adults, by age group, sex^†^, and county-level characteristics^§^ — United States, December 14, 2020—May 22, 2021

Characteristic	Vaccine coverage									
	Overall ≥18 yrs		18–29 yrs		30–49 yrs		50–64 yrs		≥65 yrs	
	(N = 255,200,373)		(n = 53,728,222)		(n = 84,488,200)		(n = 62,925,688)		(n = 54,058,263)	
	Estimate (%)	% Difference (95% CI)	Estimate (%)	% Difference (95% CI)	Estimate (%)	% Difference (95% CI)	Estimate (%)	% Difference (95% CI)	Estimate (%)	% Difference (95% CI)
**Overall**	**56.3**	**—**	37.6	—	48.5	—	62.9	—	79.1	—
**Sex^†^**										
Female	**58.0**	**Ref**	40.4	Ref	50.2	Ref	63.8	Ref	77.5	Ref
Male	**53.4**	**−4.6 (−5.5 to −3.7)**	34.0	−6.4 (−7.8 to −5.1)	45.5	−4.7 (−5.7 to −3.6)	60.3	−3.5 (−4.4 to −2.7)	79.9	2.4 (2.1 to 2.8)
**Urban/Rural status^¶^**										
Large central metro	**56.2**	**Ref**	41.0	Ref	50.9	Ref	63.2	Ref	74.8	Ref
Large fringe metro	**58.0**	**1.7 (−7.7 to 11.2)**	40.3	−0.7 (−9.0 to 7.5)	49.8	−1.2 (−10.6 to 8.2)	64.1	0.9 (−9.3 to 11.1)	79.7	4.9 (−3.4 to 13.2)
Medium metro	**53.7**	**−2.6 (−12.3 to 7.2)**	33.3	−7.7 (−15.9 to 0.5)	44.9	−6.1 (−16.0 to 3.9)	60.4	−2.8 (−13.5 to 8.0)	79.0	4.2 (−4.4 to 12.9)
Small metro	**48.6**	**−7.6 (−18.6 to 3.3)**	28.5	−12.5 (−21.9 to −3.1)	39.6	−11.4 (−22.6 to −0.2)	54.3	−8.9 (−20.9 to 3.0)	73.8	−1.0 (−11.0 to 9.0)
Micropolitan	**45.3**	**−10.9 (−22.2 to 0.3)**	23.6	−17.4 (−27.4 to −7.3)	34.5	−16.4 (−28.3 to −4.5)	50.8	−12.4 (−24.9 to 0)	71.2	−3.6 (−13.3 to 6.1)
Noncore	**42.0**	**−14.2 (−25.1 to −3.3)**	20.1	−20.9 (−31.2 to −10.6)	29.7	−21.3 (−33.2 to −9.3)	45.8	−17.4 (−29.4 to −5.4)	65.4	−9.4 (−18.2 to −0.7)
SVI quartile**										
Low vulnerability										
<25th percentile	**59.8**	**Ref**	42.1	Ref	51.6	Ref	64.5	Ref	80.9	Ref
25th to <50th percentile	**58.0**	**−1.7 (−6.8 to 3.3)**	40.0	−2.1 (−8.2 to 4.0)	51.0	−0.6 (−7.3 to 6.2)	63.4	−1.2 (−6.4 to 4.1)	80.4	−0.4 (−3.4 to 2.5)
50th to <75th percentile	**52.2**	**−7.5 (−13.6 to −1.5)**	33.9	−8.1 (−13.7 to −2.6)	44.2	−7.4 (−13.3 to −1.5)	58.3	−6.2 (−12.9 to 0.4)	74.5	−6.4 (−13.4 to 0.6)
High vulnerability										
≥75th percentile	**46.0**	**−13.8 (−23.8 to −3.7)**	28.5	−13.5 (−22.6 to −4.5)	39.2	−12.4 (−22.2 to −2.6)	53.9	−10.6 (−22.3 to 1.0)	67.4	−13.5 (−25.9 to −1.0)
**Percent of total population unemployed^††^**										
Below median (<50th percentile)	**52.6**	**Ref**	34.7	Ref	44.1	Ref	57.4	Ref	75.2	Ref
At or above median (≥50th percentile)	**54.2**	**1.6 (−2.4 to 5.7)**	35.9	1.2 (−3.3 to 5.6)	47.0	2.9 (−1.7 to 7.5)	61.0	3.6 (−0.7 to 7.9)	76.0	0.8 (−2.7 to 4.4)
**Percent of total population uninsured^††^**										
Below median (<50th percentile)	**61.7**	**Ref**	42.7	Ref	54.7	Ref	67.8	Ref	83.0	Ref
At or above median (≥50th percentile)	**44.1**	**−17.6 (−33.8 to −1.5)**	27.2	−15.5 (−26.4 to −4.6)	36.3	−18.4 (−32.9 to −4.0)	50.2	−17.6 (−35.2 to 0.0)	66.9	−16.1 (−36.6 to 4.3)
**Percent of total population below the federal poverty level^††^**										
Below median (<50th percentile)	**58.0**	**Ref**	40.0	Ref	50.0	Ref	63.5	Ref	79.7	Ref
At or above median (≥50th percentile)	**48.0**	**−10.0 (−14.3 to −5.8)**	30.4	−9.6 (−13.4 to −5.9)	41.0	−9.0 (−13.4 to −4.5)	54.8	−8.7 (−13.6 to −3.8)	70.3	−9.3 (−14.8 to −3.8)
**Percent of total population with no computer^††^**										
Below median (<50th percentile)	**55.6**	**Ref**	37.7	Ref	48.4	Ref	62.1	Ref	77.6	Ref
At or above median (≥50th percentile)	**44.0**	**−11.7 (−17.1 to −6.2)**	24.0	−13.7 (−19.3 to −8.1)	34.2	−14.2 (−20.8 to −7.6)	49.5	−12.6 (−18.4 to −6.8)	67.7	−9.9 (−13.9 to −5.9)
**Percent of total population with a computer but no Internet^††^**										
Below median (<50th percentile)	**57.4**	**Ref**	39.5	Ref	50.0	Ref	63.5	Ref	79.0	Ref
At or above median (≥50th percentile)	**42.3**	**−15.1 (−21.4 to −8.9)**	23.6	−15.9 (−20.5 to −11.3)	33.7	−16.3 (−21.9 to −10.7)	48.7	−14.7 (−21.4 to −8.0)	65.9	−13.1 (−21.6 to −4.6)
**Percent of total population of a racial/ethnic group other than non-Hispanic White^††^**										
Below median (<50th percentile)	**51.0**	**Ref**	29.1	Ref	39.9	Ref	55.4	Ref	75.9	Ref
At or above median (≥50th percentile)	**54.3**	**3.4 (−8.0 to 14.7)**	36.8	7.7 (−1.1 to 16.6)	47.4	7.4 (−3.7 to 18.6)	61.0	5.6 (−6.5 to 17.7)	75.7	−0.1 (−12.9 to 12.6)

**Abbreviations:** CI = confidence interval; NCHS = National Center for Health Statistics; Ref = referent group; SVI = Social Vulnerability Index.

* All models exclude persons with missing state of residence (modeled overall coverage is slightly lower than shown in descriptive results).

^†^ Persons with sex reported as “unknown” (N = 1,627,296) were excluded from the table (≥18 years, n = 1,627,296; 18–29 years, n = 242,601; 30–49 years, n = 578,940; 50–64 years, n = 504,173; ≥65 years, n = 301,582).

^§^ The following jurisdictions were excluded from all county-level analyses (NCHS urban-rural, SVI, and Social Determinants of Health) due to lack of county-level vaccination data: all counties in Hawaii and eight counties in California for which total population was <20,000. Among all first doses analyzed during December 14, 2020–May 22, 2021, 5.9% were missing county data and were therefore excluded from models.

^¶^ Categories of county-level urbanicity based on the 2013 NCHS urban-rural classification scheme. https://www.cdc.gov/nchs/data_access/urban_rural.htm#2013_Urban-Rural_Classification_Scheme_for_Counties

** Fifteen elements categorized into four themes (socioeconomic status, household composition and disability, racial/ethnic minority status and language, and housing type and transportation) are included in SVI (https://www.atsdr.cdc.gov/placeandhealth/svi/documentation/pdf/SVI2018Documentation-H.pdf). Overall SVI includes all 15 indicators as a composite measure (https://www.atsdr.cdc.gov/placeandhealth/svi/fact_sheet/fact_sheet.html). One county in New Mexico was excluded because SVI ranking could not be calculated (https://www.atsdr.cdc.gov/placeandhealth/svi/index.html).

^††^ Measures of Social Determinants of Health from the American Community Survey: percentage of the total population 1) unemployed, 2) uninsured, 3) that earned an income below the federal poverty level, 4) without a computer (e.g., desktop or laptop computer [excludes mobile phones]), 5) with a computer but without Internet access, and 6) identifying as a racial/ethnic group other than non-Hispanic White (https://health.gov/healthypeople/objectives-and-data/social-determinants-health).

## Discussion

As of May 22, 2021, COVID-19 vaccination coverage among U.S. adults was highest among adults aged ≥65 years and lowest among adults aged 18–29 years. Despite recently expanded eligibility for vaccination to all adults, increases in weekly initiation among younger age groups have not reached peak weekly initiation rates that occurred in January and February among adults aged ≥65 years. If the current rate of weekly vaccine initiation continues through August, coverage among young adults will not reach the coverage level of older adults. High vaccination coverage among all age groups is important for decreasing COVID-19 cases, hospitalizations, and deaths (*2*,*3*), especially among groups with lower vaccination uptake, such as young adults (*4*,*5*).

Equitable access to vaccination is critical to improve coverage for persons of all ages who live in communities that are less urban (*6*), have higher social vulnerabilities (*1*,*7*), and have higher percentages of social determinants of poor health (*8*). In a report that pooled findings from two representative surveys of U.S. adults aged 18–39 years, only one half (51.8%) reported that they had been or were planning to be vaccinated, whereas 24.9% reported that they probably or definitely would not be vaccinated, and 23.2% reported that they would probably be vaccinated or were unsure if they would be vaccinated (*9*). Respondents who were reluctant or unsure about vaccination reported concerns about vaccine side effects, distrust of COVID-19 vaccines, a plan to wait and see whether the vaccine was safe and to possibly get vaccinated later, thinking that others needed a vaccine more than they did, and the belief that they did not need the vaccine. Low intention to receive COVID-19 vaccination among younger adults aligns with historic vaccination coverage for influenza^†††††^ and lower adherence to COVID-19 public health guidelines (*10*). For coverage among persons in this age group to be improved, community-specific messaging could engage younger adults using trusted sources to explain the community and individual value of vaccination and to address concerns about vaccine safety. In addition, younger adults might be reached by establishing strategically located mobile and walk-in clinics with flexible hours,^§§§§§^ providing vaccinations at the workplace, and encouraging employers to offer paid leave for employees to receive the vaccine and for treatment of any vaccine-related side effects.^¶¶¶¶¶^

The findings in this report are subject to at least four limitations. First, general periods were used that applied broadly to eligibility periods for most states; however, states varied in their expansion of vaccine eligibility over time, thus vaccine initiation by age might differ if evaluated using precise eligibility periods. Second, the ecologic findings for vaccination coverage by community-level factors do not reflect the status of individual persons. Third, county-level characteristics might vary at a smaller geographic level; future analyses could consider using a more granular assessment of community factors that are associated with poor health. Finally, coverage might be underestimated because persons for whom county of residence were incomplete were excluded from models.

Despite expanded eligibility to all adults in the United States by April 19, 2021, vaccine initiation among persons aged <65 years has not increased at the same rate observed in earlier periods among persons aged ≥65 years. Continued targeted efforts are needed to accelerate vaccination rates, especially among younger adults. Community-based outreach efforts to increase vaccine confidence and reduce potential barriers to access could improve COVID-19 vaccination initiation, particularly among persons aged 18–29 years, and reduce the spread and impact of COVID-19 among the general U.S. population.

**Summary**

**What is already known about this topic?**

The U.S. COVID-19 vaccination program initially prioritized groups at highest risk for COVID-19 hospitalization and death; by April 19, 2021, eligibility expanded to all persons aged ≥16 years.

**What is added by this report?**

By May 22, 2021, 57.0% of U.S. adults aged ≥18 years had received ≥1 vaccine dose; coverage was lower and increased more slowly over time among younger adults. If the current rate of vaccination continues through August, coverage among young adults will remain substantially lower than among older adults.

**What are the implications for public health practice?**

Efforts to improve vaccination coverage are needed, especially among younger adults, to reduce COVID-19 cases, hospitalizations, and deaths.

## Footnotes

*
https://www.cdc.gov/mmwr/volumes/69/wr/mm695152e2.htm

† Dates of vaccine eligibility opened to persons aged ≥16 years based on data from the Kaiser Family Foundation State COVID-19 Data and Policy Actions (https://www.kff.org/report-section/state-covid-19-data-and-policy-actions-policy-actions/).

§ Immunization information systems are confidential, computerized, population-based systems that collect and consolidate vaccination data from providers in 64 jurisdictions nationwide and can be used to track administered vaccines and measure vaccination coverage. The 64 jurisdictions comprise the 50 U.S. states, five U.S. territories (American Samoa, Guam, Northern Mariana Islands, Puerto Rico, and U.S. Virgin Islands), three freely associated states (Federated States of Micronesia, Marshall Islands, and Palau), and six local jurisdictions (Chicago, Illinois; Houston, Texas; New York, New York; Philadelphia, Pennsylvania; San Antonio, Texas; and Washington, DC).

¶
https://www.cdc.gov/vaccines/covid-19/reporting/vams/program-information.html

**
https://www.cdc.gov/vaccines/covid-19/reporting/overview/IT-systems.html

†† Vaccination providers are required to report administration records to the state immunization information system within 72 hours; 5 additional days of observation were included to account for delays in reporting and transmission of records to CDC.

§§
https://www.census.gov/programs-surveys/popest.html

¶¶ An epidemiologic week is based on the National Notifiable Diseases Surveillance System guidance and is assigned by the reporting local or state health department for the purposes of *MMWR* disease incidence reporting and publishing. The first day of any *MMWR* week is Sunday. https://wwwn.cdc.gov/nndss/document/MMWR_Week_overview.pdf

*** Analysis for second dose completion was restricted to persons who had received their first dose of a 2-dose vaccine (Pfizer-BioNTech or Moderna) during December 14, 2020–March 31, 2021. All persons included in the analysis for second dose completion were ≥42 days past their first dose.

††† Periods are based on eligibility and other process factors (e.g., phase of vaccine rollout, eligible population, supply, and programs and policy enacted) important in framing the specific needs and constraints at that time. Period 1 represented when most states opened eligibility to health care workers, residents in long-term care facilities, and older adults while there was a highly constrained supply, which overlapped phase 1a, and a portion of phase 1b (https://www.cdc.gov/mmwr/volumes/69/wr/mm695152e2.htm). Period 2 represented when states were expanding eligibility inconsistently, and supply was becoming more available, which overlapped with phases 1b and 1c. Period 3 represented when all states expanded eligibility to all adults while supply was steady and increased, which overlapped with phases 1c and 2.

§§§ The following jurisdictions were excluded from all county-level analyses (National Center for Health Statistics urban-rural, SVI, and Social Determinants of Health) due to lack of county-level vaccination data: all counties in Hawaii and eight counties in California for which total population was <20,000. Among all first doses analyzed during December 14, 2020–May 22, 2021, 5.9% were missing county data and were therefore excluded from models.

¶¶¶ Fifteen elements categorized into four themes (socioeconomic status, household composition and disability, racial/ethnic minority status and language, and housing type and transportation) are included in SVI (https://www.atsdr.cdc.gov/placeandhealth/svi/documentation/pdf/SVI2018Documentation-H.pdf). Overall SVI includes all 15 indicators as a composite measure (https://www.atsdr.cdc.gov/placeandhealth/svi/fact_sheet/fact_sheet.html). One county in New Mexico was excluded because SVI ranking could not be calculated (https://www.atsdr.cdc.gov/placeandhealth/svi/index.html).

**** Measures of Social Determinants of Health from the American Community Survey: percentage of the total population 1) unemployed, 2) uninsured, 3) that earned an income below the federal poverty level, 4) without a computer (e.g., desktop or laptop computer [excludes mobile phones]), 5) with a computer but without Internet access, and 6) identifying as a racial/ethnic group other than non-Hispanic White (https://health.gov/healthypeople/objectives-and-data/social-determinants-health).

††††
https://www.census.gov/programs-surveys/acs

§§§§ In some instances, the total non-Hispanic White population exceeded the total population estimate and therefore the model did not permit vaccination initiation estimates to exceed 100%.

¶¶¶¶
https://www.cdc.gov/nchs/data_access/urban_rural.htm#2013_Urban-Rural_Classification_Scheme_for_Counties

***** 45 C.F.R. part 46, 21 C.F.R. part 56; 42 U.S.C. Sect. 241(d); 5 U.S.C. Sect. 552a; 44 U.S.C. Sect. 3501 et seq.

††††† Estimates from the Behavioral Risk Factor Surveillance System and the National 2009 H1N1 Flu Survey (https://www.cdc.gov/flu/fluvaxview/trends/age-groups.htm).

§§§§§ Mobile Vaccination Resources (https://www.cdc.gov/vaccines/covid-19/planning/mobile.html); Key Operational Considerations for Jurisdictions Planning to Operate COVID-19 Vaccination Clinics (https://www.cdc.gov/vaccines/covid-19/downloads/Key-Op-Considerations-COVID-Mass-Vax.pdf).

¶¶¶¶¶
https://www.whitehouse.gov/briefing-room/statements-releases/2021/04/21/fact-sheet-president-biden-to-call-on-all-employers-to-provide-paid-time-off-for-employees-to-get-vaccinated-after-meeting-goal-of-200-million-shots-in-the-first-100-days/
